# The sense of agency in autism spectrum disorders: a dissociation between prospective and retrospective mechanisms?

**DOI:** 10.3389/fpsyg.2015.01278

**Published:** 2015-09-08

**Authors:** Tiziana Zalla, Marco Sperduti

**Affiliations:** Department of Cognitive Studies, Ecole Normale Supérieure, Institut Jean Nicod, Centre National de la Recherche Scientifique, Paris Sciences et Lettres Research University, Paris, France

**Keywords:** action monitoring, comparator model, intentional binding, intrapersonal cognition, metacognition, agency

## Abstract

While a large number of studies have reported impairments in social and interpersonal abilities in individuals with autism spectrum disorder (ASD), relatively few studies have focused on self-related knowledge in this population. One of the processes implicated in the physical dimension of the Self is the sense of agency (SoA), i.e., the experience of initiating and controlling one’s own actions and producing desired changes in the world via these actions. So far, the few studies investigating SoA in ASD have reported contrasting results, with some showing spared, others impaired SoA. Here, we review the existing literature and suggest that the distinction between prospective and retrospective mechanisms of the SoA might help reconcile the existing findings. In the light of a multi-componential model of SoA, we propose the view that a specific impairment at the level of *prospective* mechanisms acting on internal agency signals (i.e., the intention, action selection, or command produced to achieve the goal) may be responsible for the reduced SoA in ASD, along with spared retrospective mechanisms. Future research should shed light on the impact of abnormal SoA on social and self-related dysfunctions in ASD.

## Introduction

Autism spectrum disorders (ASD) are characterized by social and communicative impairments, restricted interests, and repetitive behaviors (American Psychiatric Association, 2013). More recently, research has provided circumstantial evidence suggesting that some forms of intrapersonal cognition might be altered in people with ASD (Uddin, 2011), and that diminished self-related knowledge might be crucially linked to early childhood social impairments in this population (Lombardo et al., 2010). However, while abundant research has investigated social and communication impairments in individuals with ASD, intrapersonal and self-representation processes have received little attention.

One fundamental distinction posited by theoretical models of self-representation is that between implicit and explicit processes (Klein and Gangi, 2010). The former mainly involve body-grounded mechanisms, such as proprioceptive and sensorimotor processes, whereas the latter typically implicate abstract self-knowledge and autobiographical memory (e.g., Conway, 2005). Within the Self-Memory System, impaired episodic memory along with spared semantic autobiographical memory and self-trait knowledge have been reported in ASD (Lind, 2010), and Lombardo et al. (2010) have shown atypical processing of abstract self-knowledge in individuals with ASD. In line with these findings, it has been proposed that declarative processes related to the Self are impaired (Lind, 2010; Uddin, 2011), while physical and embodied aspects of the Self, such as the sense of agency (SoA), are relatively preserved in this population (David et al., 2008a; Williams and Happé, 2009). However, previous evidence on SoA in individuals with ASD using different experimental paradigms has brought mixed results, with studies showing either preserved (David et al., 2008a; Williams and Happé, 2009) or impaired SoA (Grynszpan et al., 2012; Sperduti et al., 2014; Zalla et al., 2015).

Theoretical proposals have been advanced to explain the SoA in normal and pathological conditions, with some accounts emphasizing prospective mechanisms occurring prior to action execution (e.g., Chambon et al., 2014), and others assigning a preponderant role to retrospective mechanisms occurring after action execution (e.g., Wegner et al., 2004). These two types of account are not necessarily contradictory, but while prospective and retrospective processes can be distinguished in experimental settings, they are usually integrated in more ecological and everyday life conditions. Recently, Moore and Fletcher (2012) suggest that internal (motoric) and external (reafferent sensory information) signals, that in our framework roughly correspond to prospective and retrospective mechanisms respectively, are weighted, depending on their availability and their reliability, to produce a coherent SoA.

To clarify this controversial issue, we propose here that a distinction needs to be made between different types of component processes contributing to the SoA, and that apparently contradictory results can be conciliated by postulating a dissociation between impaired prospective and spared retrospective processes of the SoA in ASD. Given the lack of a general consensus on the mechanisms which are specifically involved in the genesis of SoA, we will here use the term *prospective* to refer to those processes occurring before overt action execution and we will use the term *retrospective* to denote those processes occurring after action execution. The latter includes comparator mechanisms, as well as higher-level inferences based on motor performance (e.g., judgment of performance) or contextual information.

In the following, we will first present an overview of the existing models of the SoA based on empirical studies in typical population and individuals with ASD, and then review their implication for our theoretical proposal.

## Sense of Agency: A Multi-Componential Account

The SoA is the experience of initiating and controlling one’s own action and hence producing desired changes in the world through these actions (Haggard and Tsakiris, 2009). As such, it is a fundamental ability grounding all kinds of efficient self-world interactions, from instrumental actions to social exchanges. The SoA refers to a complex cognitive phenomenon; in everyday life, it is experienced as a “diffuse sense of a coherent, harmonious on-going flow of action processing” (Synofzik et al., 2008, p. 228).

The predominant theory explaining the SoA is based on the Central Monitoring mechanism and, in particular, the *Comparator Model* (Wolpert et al., 1995; Frith et al., 2000). Initially developed to account for sensory-motor control (e.g., Wolpert and Miall, 1996), the Comparator Model was subsequently extended to a model of the SoA (Frith et al., 2000). It states that the sensory consequences of one’s behavior can be predicted based on internal action-related information, such as the efferent copy of a central motor command (Bell, 2001). While the efferent copy is used by the forward model to predict the state of the system, the afferent sensory inputs are used to estimate the actual state. If predicted and estimated actual states are congruent, the actions are experienced as self-performed whereas, in case of a mismatch, incongruent signals indicate either an erroneous prediction or an external source as the cause of that action. Hence, the matching process between central motor commands and feedback signals arising during action execution is the crucial mechanism underlying the SoA. Importantly, this implies that the emergence of a SoA can be inferred retrospectively after action execution, that is only after reafferent sensory signals are processed and compared with the “internal prediction.” In this view, the SoA is regarded as a retrospective inference concerning the action-effect causal structure.

A more radical retrospective account of SoA posits that the actual execution of voluntary action is not even necessary to experience agency, but that the co-occurrence of outcomes that are coherent with the agent’s prior intentions would be sufficient for the emergence of this experience (see Wegner et al., 2004). While this account assigns to the comparator mechanisms a central role in generating SoA retrospectively, alternative theories provide support for the view that prospective mechanisms also play a pivotal role in the emergence of a SoA, and crucially contribute to the generation of the subjective feeling of control over the action outcome (Moore and Haggard, 2008; Chambon et al., 2014).

However, a consensual view on the nature and the role of the prospective processes involved in generating SoA is still lacking. People use a variety of cues to assign agency, but how this information dynamically interacts to form the unitary feeling of consciously willing the action is still a matter of debate. In a recent review, Hughes et al. (2013) have suggested that predictions can be made about the motor identity of the stimulus, based on the performed action (motor identity prediction), or about the timing of a sensory stimulus (temporal prediction). While neither process seems necessary to produce SoA, the mere presence of an action can drive this phenomenon, suggesting the involvement of the motor system. Nevertheless, as the authors also underlined, existing studies do not include the appropriate experimental conditions to effectively evaluate the differential impact of various predictive signals. Moreover, other processes occurring before action execution and that are unrelated to predictive mechanisms, such as action selection, may contribute to SoA (e.g., Chambon et al., 2014). Hence, the broader category of *prospective* processes is used to denote all mechanisms occurring before action execution, whereas retrospective mechanisms refer to all processes occurring after action execution.

To clarify the contribution of prospective and retrospective processes to action awareness and to the SoA, Moore and Haggard (2008) employed an experimental paradigm based on intentional binding (IB), an implicit measure of the SoA (Haggard et al., 2002). IB consists in the temporal attraction between a voluntary operant action and its effect (e.g., a tone). In this experiment, participants were instructed to press a key at a time of their own choosing which caused an effect (e.g., a tone) 250 ms later. In one condition, 75% of the actions were followed by a tone; in the other condition, only 50% of the actions caused a tone. The authors found that when the action-effect contingency was highly predictable (as in the 75% probability condition), IB was present even in the absence of the outcome, when no matching process was possible, suggesting that in this condition prospective cues drive SoA. Conversely, when the probability was low (as in the 50% probability condition), the IB only occurs when the outcome was actually present. These findings suggested that both prospective and retrospective mechanisms contribute to SoA.

More recently, Moore and Fletcher (2012) have proposed that SoA results from the integration of multiple available cues within a *Bayesian* model that combines prior knowledge or expectations operating as prospective cues with sensory data acting as retrospective cues. The action-effect relation can be considered as *prior knowledge*, built up by inferring the causal structure from patterns of statistical correlation over the course of the block, whereas the various sources of information including efferent and somatosensory information, as well as the auditory information about the tone following the action are regarded as *sensory data*. On this view, the SoA involves the integration not only of multiple signals from a single event, but also the integration of predictions, built up over the course of previous actions, with information from sensory events on the current trial (Moore and Fletcher, 2012). Thus, prospective and retrospective information are weighted, depending on their availability and their reliability, to produce a coherent SoA.

It is important to note that previous reports on normal and pathological conditions have usually employed experimental paradigms tapping the ability to recognize actions as being self-generated or generated by external agents, in which prospective and retrospective signals contributing to the SoA were conflated or not adequately dissociated (David et al., 2008b). Action recognition studies in patients with parietal lobe lesions have shown that failure to detect the mismatch between sensorimotor afferent information and visual feedback about self-performed movements is not sufficient to generate abnormal SoA (Sirigu et al., 1999), and research on amputees has revealed that, in the absence of reliable proprioceptive or sensorimotor cues, other sources of information contribute to SoA (Ramachandran and Rogers-Ramachandran, 1996). Importantly, prospective and retrospective processes can be selectively impaired, as already shown in patients with schizophrenia (Voss et al., 2010).

## The Sense of Agency in ASD

The “Comparator Model” posits that action monitoring is a central mechanism for the emergence of SoA. Within this framework, impairment at the level of action monitoring is often taken as indirect evidence of SoA disruption. Pioneer studies by Russell and Jarrold (1998, 1999) suggested that an impaired mechanism relating motor commands to their visual outcomes might underlie diminished action monitoring and SoA in ASD. The authors employed a task in which children with and without autism had to choose, by pressing a left or right key, which of two characters would serve a ball to hit a target that appeared either to the left or to the right (Russell and Jarrold, 1998). In half of the trials, the task generated a stimulus-response incompatibility provoking errors, and subjects had the possibility to correct their error pressing the opposite button. The results showed that children with ASD made more errors and corrected a lower proportion of wrong answers, suggesting an action monitoring impairment. In a subsequent study, Russell and Jarrold (1999) reported that children with ASD had difficulties in correctly deciding whether an action had been produced by themselves or by another agent. In line with these findings, lack of self-reference (Toichi et al., 2002) and reduced memory enhancement for self-performed, as compared to others’ (visually encoded) actions, have been reported in adults with high functioning ASD (Zalla et al., 2010; Daprati et al., 2013). Various interpretations have been offered for this failure, including an impaired mechanism relating action motor commands to their visual outcomes (Russell and Jarrold, 1999; Zalla et al., 2010), a strong dependence on the increased executive demands produced by the task (Hala et al., 2005) or a delayed development of source monitoring abilities, which would be strictly dependent on verbal mental age (Farrant et al., 1999).

However, subsequent studies failed to replicate these findings. For example, Hill and Russell (2002) did not observe difficulties in self-other attribution of previously executed actions in children with ASD. Russell and Hill (2001) showed that children with ASD were as able as the control group in discriminating their own actions from those of an external agent by judging on-line which one of several colored dots presented on a computer screen was under their intentional control (through movements of the mouse). Similarly, Williams and Happé (2009) found that children with ASD had no difficulties monitoring their own actions/agency using an on-line action monitoring task requiring individuals to distinguish person-caused from computer-caused changes in visually presented squares. A study by David et al. (2008a) directly investigated the SoA in adults with ASD using a target completion task. Participants had to move a cursor on a computer screen, controlled by a joystick, toward one of two targets and could track the trajectory of their movements on the screen. At the end of each trial they were asked to judge whether the visual feedback matched the performed movement and whether this was self-generated or not. The task manipulated the degree of correspondence between the participants’ movements and the corresponding visual feedback. Unbeknownst to the participants, in 50% of the trials, they received a false visual feedback for the path of the cursor. The authors reported that participants with and without ASD did not differ in their accuracy in judging self-other agency, and concluded that agency and action monitoring were preserved in ASD. Indeed, these findings seem to indicate that motor identity prediction, and the comparison with reafferent visual signals are spared in ASD. In addition, it is important to note that, in this experiment, the action was followed by the actual feedback in 50% of the trials, making the actual movements not reliably predictive of the visual feedback. The participants were explicitly asked to report whether the visual feedback matched their movement, making it possible that the assignment of agency in participants with ASD would rely retrospectively on the comparator mechanism. Alternatively, since in the actual feedback conditions reaching the target might be easier, as compared to the distorted feedback conditions, the assignment of agency could also be inferred retrospectively on the basis of external cues, such as the subjective goodness of performance.

In accordance with this interpretation, Zalla et al.’s (2015) findings support the hypothesis of intact retrospective mechanisms for the SoA in ASD. In this study, participants were asked to perform a computer-based task in which they had to touch Xs and avoid touching Os streaming down the screen by moving a box on a gray track via the computer mouse. The paradigm allowed investigating the influence of the objective manipulation of control on the judgment of agency in individuals with ASD. In addition to the condition in which the box displacements were totally under the participants’ control, there was an experimental condition in which participants’ control of the cursor was altered by the introduction of a time lag between mouse and cursor movement, or by the addition of turbulence (random noise) to the cursor position. At the end of each trial, participants judged their own control over the game (i.e., made a judgment of agency) and how successful they were at touching the Xs and not touching the Os (i.e., made a judgment of performance). The results revealed a decrement in the actual motor control, together with a good accuracy level in their metacognitive judgment of performance (i.e., the perceived goodness of performance) and judgment of agency. However, while all participants grounded their judgment of agency on judgment of performance, participants with ASD showed reduced sensitivity to and diminished use of the internal sensorimotor cues generated during the experimental manipulations. These diagnostic cues are responsible for forming an internal prediction model (i.e., the efferent copy) of self-performed action, likely affecting both action monitoring and SoA in ASD.

These findings have two important implications. First, subtle impairments in SoA and an altered sense of control might be observed in participants with ASD when the SoA is not operationalized as an all-or-nothing process (presence or absence of SoA), but rather as a continuum of a variable subjective strength of control. Second, in line with our proposal, the SoA in ASD relies mostly on a preserved metacognitive judgment of agency, based on retrospective signals, while it might be affected by a reduced reliance on predictive sensorimotor cues or by atypical forms of intermodal weighting of action signals. Hence, when asked to evaluate their action performance and control, individuals with ASD would tend to rely more on retrospective processes (judgments of performance), than on actual control. In making their judgments of agency, they used external cues, such as goodness of performance, but they relied to a lesser extent than did control participants on the particular internal sensory motor cues that are diagnostic of agency.

Recently, we have investigated SoA in a group of adults with ASD using the IB paradigm (Sperduti et al., 2014). In our task, the action effect contingency was highly reliable (the action was always followed by the same stimulus –100% contingencies). Importantly, under this condition, when the action-effect association is reliable, predictive cues have been shown to drive IB and SoA (Moore and Haggard, 2008). The main results showing that IB was reduced—but not completely abolished—in adults with ASD are in line with the hypothesis of altered prospective mechanisms in this population. Further studies are needed to directly test this hypothesis, since our experimental manipulation did not permit a straightforward distinction between prospective and retrospective processes.

In line with our interpretation, “Bayesian cue integration models” of agency posit that the SoA relies on the weighted integration of multiple internal and external agency cues together with prior beliefs. Recently, it has been suggested that both social and non-social impairments in ASD could be accounted for by an abnormal weighting of prior expectations and sensory information, possibly resulting from abnormally high sensory precision and enhanced bottom-up functioning (Pellicano and Burr, 2012; Lawson et al., 2014). Specifically, the attenuation of Bayesian priors—“hypo-priors”—may be responsible for the atypical sensory experience and perception of the world, which would be more perceptually accurate and less modulated by prior experience in individuals with ASD. It is thus likely that such abnormal interplay between bottom-up sensory signals and top-down predictions, based on abstract prior knowledge and background information, might also explain the atypical or reduced SoA in this population.

In a recent study, Chambon and Haggard (2012) showed that SoA in typical populations might also be informed by early signals generated at the moment of action selection. In these circumstances, the source of the prospective information is the action selection mechanism, which operates prior to action performance and to action–outcome matching. Following the authors’ proposal, the signals relating to the fluency of action selection (on the so-called “Inverse model”) are temporally stored in an *Intentional Buffer* and prospectively generate the SoA during the process of action planning. Action fluency signals arising from action selection processes may operate, at an implicit level, to produce a feeling of control, and be buffered until sensorimotor feedbacks ensuring the matching process are available. At the conscious level, this prospective experience of agency might constitute the basis for the process of self-attribution of intention. An abnormal sense of action control and SoA in people with ASD might indeed reflect limited accessibility to prospective internal agency cues based on action fluency selection, at the level of action planning.

Converging evidence suggests that participants with ASD have difficulties introspecting their own motor intentions (Phillips et al., 1998; Williams and Happé, 2010), and that the earlier stages of action planning and movement preparation might be impaired (Rinehart et al., 2001, 2006; Dowd et al., 2012; Daprati et al., 2013). As previously reported (Williams and Happé, 2010), children with ASD were less able to recognize their own knee-jerk reflex movements as unintentional, or their own mistaken actions as unintended, than age- and ability-matched comparison participants, which suggests a diminished awareness of their own intentional states. It is worth noting that, in Russell and Jarrold’s (1998) aforementioned study, children with ASD were slower in giving correct answers, but they were as fast as the control group when they succeeded in correcting their errors. While these results were taken as reflecting an action-monitoring impairment associated with a disrupted CM mechanism, an alternative interpretation is that slower reaction times in choosing the correct answer might indeed reflect difficulties in action selection or action fluency. In assigning agency, individuals with ASD would fail to adequately combine prospective cues, based on action selection or action fluency, with retrospective proprioceptive or visual feedbacks.

**FIGURE 1:**
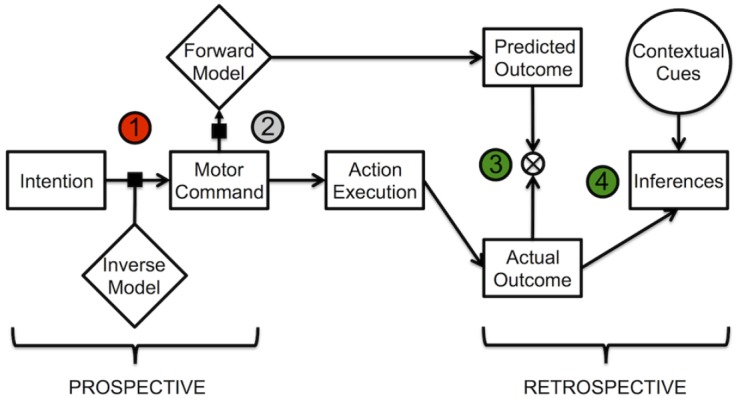
**Schematic representation of mechanisms involved in SoA.** Impairment of SoA could emerge at the level of (1) action selection; (2) predictive processes implemented by the forward model; (3) comparator mechanisms, and (4) inferential processes based on actual motor performances (e.g., judgment of performance) or contextual cues. Red circle (1) represents likely impaired mechanisms leading to altered SoA in ASD; gray circle (2) represents processes for which there is mixed evidence, green circles (3, 4) represent likely spared processes in ASD.

Taken together, these findings support the view that multiple prospective and retrospective cues contribute to the creation of a reliable SoA, with little explicit knowledge available to the agent concerning how this integration process is computed by the brain. Importantly, the absence of or the limited sensitivity to early prospective agency cues associated with voluntary action (i.e., fluency of action selection, or command produced to achieve the goal) could determine an abnormal SoA and an impairment in the self-attribution of intentions. While in normal conditions, both external and internal sources of information are used to determine the SoA, in individuals with ASD the influence of retrospective cues may increase when the reliability or accessibility of internal agency signals decreases. Even if speculative, this hypothesis can be tested by employing paradigms that allow distinguishing the specific contribution of prospective and retrospective mechanisms to the SoA. One example is the manipulation employed in the aforementioned study by Moore and Haggard (2008) which provided convincing evidence of a similar dissociation in schizophrenia. Other possibilities are offered by neuroimaging techniques (fMRI, EEG). Indeed, if prospective mechanisms were selectively impaired in ASD, we would expect to find functional abnormalities in regions underpinning action planning and SoA, such as the angular gyrus, the premotor and supplementary motor areas (Chambon et al., 2013; Moore et al., 2010; Sperduti et al., 2011), before action execution. Figure 1 schematically represents the putative mechanisms involved in SoA, and the different stages at which the impairment might occur in ASD.

## SoA as a Precursor of Social Impairment in ASD?

In accordance with the theoretical accounts of the SoA which distinguishes prospective and retrospective agency cues (e.g., Chambon et al., 2014), we have reviewed convergent evidence supporting the notion that ASD is characterized by impaired prospective and spared retrospective processes underlying the SoA. Specifically, we have argued that the distinction between the different sources of information generating the SoA allows explaining divergent results on the SoA in ASD. Previous studies reporting spared SoA in ASD employed tasks that tapped more on retrospective cues (e.g., David et al., 2008a), or on retrospective metacognitive judgments of agency (e.g., Zalla et al., 2015), whereas diminished SoA can be observed when the task maximized reliance on prospective mechanisms (Sperduti et al., 2014). Hence, we have suggested that the impairment might be characterized by reduced reliance on some critical prospective agency signals generated at the earlier stage of action selection and planning, as explained by the *Inverse Model*. While prospective and retrospective signals are normally combined to generate the SoA, less reliance on internal prospective signals in ASD might, in turn, be responsible for an atypical form of intermodal weighting of agentic action signals, resulting in a diminished SoA, and likely in impairment in self-attribution of intentions. Although an extensive literature has largely documented disturbances of mindreading or Theory of Mind (ToM), i.e., the ability to attribute beliefs and other mental states to oneself and to others in individuals with high functioning ASD (Bowler, 1992; Happé, 1994; Baron-Cohen et al., 1999; Zalla et al., 2009), fewer studies have investigated the relationship between mindreading and intrapersonal cognition, such as SoA, and in this population. Interestingly, in Zalla et al. (2015), reduced sensitivity to endogenous sensorimotor activity correlated with difficulties in an advanced ToM task in participants with ASD, suggesting a close link in development between SoA and mindreading.

Previous studies investigating SoA using social scenarios have shown that when two typically developed individuals are engaged in bringing about a common outcome, a “we” identity is automatically and pre-reflectively formed, and both partners register agency at the pre-reflective level (Obhi and Hall, 2011). Pfister et al. (2014) further extended these results by showing that when the action of one participant (the *leader*) produces a stimulus that serves as an imperative signal for the action of a second participant (the *follower*), the *leader* implicitly experiences SoA not only for her action’s effect, but also for the *follower*’s action. These findings suggest that we not only feel control over changes that we produce in the physical world, but that our agency experience might extend to the changes produced in other agents’ behavior in a social context.

Taken together, these results highlight the role of low level predictive mechanisms as building blocks for higher level cognitive functions, and open up new paths for the understanding of the relationship between abnormal SoA and social impairments in ASD.

### Conflict of Interest Statement

The authors declare that the research was conducted in the absence of any commercial or financial relationships that could be construed as a potential conflict of interest.
